# The electrophotonic silicon biosensor

**DOI:** 10.1038/ncomms12769

**Published:** 2016-09-14

**Authors:** José Juan-Colás, Alison Parkin, Katherine E. Dunn, Mark G. Scullion, Thomas F. Krauss, Steven D. Johnson

**Affiliations:** 1 The Department of Electronics, University of York, Heslington, York YO10 5DD, UK; 2 Department of Physics, University of York, Heslington, York YO10 5DD, UK; 3 Department of Chemistry, University of York, Heslington, York YO10 5DD, UK

## Abstract

The emergence of personalized and stratified medicine requires label-free, low-cost diagnostic technology capable of monitoring multiple disease biomarkers in parallel. Silicon photonic biosensors combine high-sensitivity analysis with scalable, low-cost manufacturing, but they tend to measure only a single biomarker and provide no information about their (bio)chemical activity. Here we introduce an electrochemical silicon photonic sensor capable of highly sensitive and multiparameter profiling of biomarkers. Our electrophotonic technology consists of microring resonators optimally *n*-doped to support high Q resonances alongside electrochemical processes *in situ*. The inclusion of electrochemical control enables site-selective immobilization of different biomolecules on individual microrings within a sensor array. The combination of photonic and electrochemical characterization also provides additional quantitative information and unique insight into chemical reactivity that is unavailable with photonic detection alone. By exploiting both the photonic and the electrical properties of silicon, the sensor opens new modalities for sensing on the microscale.

## Abstract (web summary)

A label-free and low-cost sensor capable of detecting several biomarkers in parallel is a sought-after medical tool. Here, the authors describe an approach exploiting both photonic and electrochemical properties of silicon to build a multi-domain biosensor for label-free and multiplexed detection.

## Graphical abstract

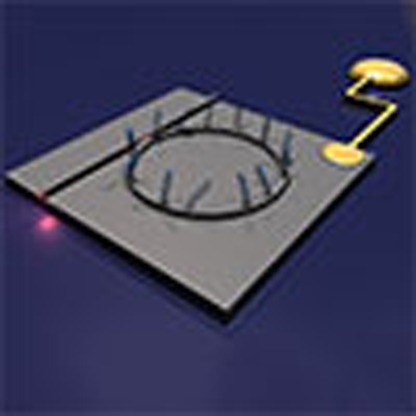

The complexity and heterogeneity of many diseases, and their dependence on lifestyle and genetics, demand new *in vitro* diagnostic technology that can provide quantitative measurement of multiple disease biomarkers in real time and at low cost. These challenges have been partly addressed by a range of label-free diagnostic platforms, many based on optical^1^,^2^ or electrochemical^3^,^4^,^5^ transduction of a biological event. Resonant silicon photonic devices such as microrings^6^,^7^,^8^ and photonic crystal cavities^1^,^9^,^10^ have been demonstrated to have considerable potential, as they offer high sensitivity, label-free detection in a format that can be mass-manufactured and have been commercialized successfully^11^. The critical property of silicon, which is the key to its widespread use in photonic biosensors, is its high refractive index. Somewhat surprisingly, the ability to tune the electrical properties of silicon in order to combine the complementary information revealed by electrochemical and photonic sensing in a single platform has not yet been explored. In part, this is due to the apparent conflict between the optimal material properties required by electrochemical and photonic sensors; electrochemistry requires materials of high conductivity, that is, high doping density, while ideal photonic materials have a low doping density in order to minimize free carrier losses. For example, a doping density of *n*≈10^18^ cm^−3^, which leads to a moderate conductivity of ≈45 S cm^−1^, is already sufficient to limit the Q-factor (resonance frequency divided by the spectral width at half maximum) to ∼10,000—much lower than the typical values of 40,000–140,000 associated with microring resonator sensors^7^,^8^,^12^,^13^.

In this paper, we present a solution to this incompatibility by controlling the doping density profile of a silicon photonic microring resonator such that the dopants are located in a thin layer at the silicon surface. The doped surface layer can thus be optimized to be sufficiently conductive to support electrochemical processes while thin enough to minimize losses of the optical mode confined within the resonant structure. This is validated by optical measurements that show that the Q-factor of the resonant structure is only marginally diminished as a result of doping, enabling highly sensitive measurement of a surface-tethered molecular layer. Furthermore, the high doping level confined to the silicon surface allows us to simultaneously characterize the electrochemical activity of a surface-immobilized redox-active monolayer, leading to an unambiguous quantitative measure of molecular surface density.

The inclusion of electrochemical characterization alongside photonic sensing not only allows us to combine the complementary information contained within the two measurement domains, but also to exploit electrochemical processes to selectively modify the silicon surface. We use this capability to electrochemically graft specific molecular probes on the sensor surface, focusing here on single-stranded DNA probes. Critically, the electrografting reaction is spatially localized, enabling site-selective control over the surface chemistry. We validate this site selectivity by demonstrating a multiplexed electrophotonic sensor array in which each microring within the array is selectively functionalized with a different probe molecule, and which is capable of monitoring multiple biomarkers in parallel.

## Results

### Electro-optical ring resonator sensor

**Figure 1:**
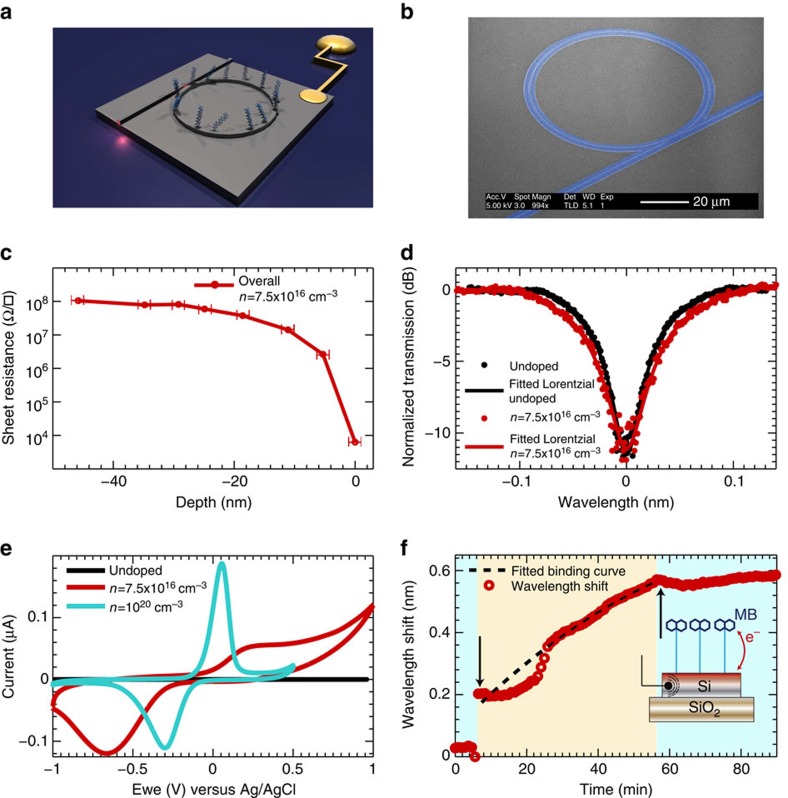
Electro-optical ring resonator biosensor. (**a**) 3D sketch of a single electro-optical device. Ohmic contacts fabricated on the doped silicon substrate allow electrochemical control over the sensor surface. (**b**) False coloured electron micrograph of the electro-optical device. The ring cavity and access waveguide are presented in blue, while the non-lithographed substrate is grey. (**c**) Sheet resistance of the device as a function of depth into the *n*-type doped silicon layer of the device. The error bars represent the systematic error in the depth measurement performed using ellipsometry. (**d**) Resonance dip of a doped device compared with that of a microring resonator of identical geometry but fabricated on undoped silicon, showing a moderate broadening due to the increase in optical loss. The resonance wavelengths were 1,580.2 and 1,580.17 nm for the undoped and doped substrates, respectively. (**e**) Cyclic voltammograms corresponding to MB-modified photonic electrodes fabricated in silicon substrates of variable surface-doping density. Electrochemical measurements were performed in a three-electrode configuration using an Ag/AgCl reference electrode and platinum counter electrode with a 100 mM potassium phosphate buffer pH 7 at a voltage scan rate of 50 mV s^−1^. The positive and negative current peaks observed using highly doped silicon (*n*=10^20^ cm^−3^) are indicative of electrochemical oxidation and reduction of MB. Similar redox current peaks are also observed at a lower average doping density of *n*=7.5 × 10^16^ cm^−3^, indicating sufficiently high carrier density in the doped surface layer to support electrochemical interrogation and control of redox molecules on the silicon surface. The increased separation between the oxidation and reduction peaks (red curve) compared with the highly doped silicon (turquoise curve) reflects the differences in carrier depletion and at electrode–solution interface. Undoped silicon (black curve) does not function as an electrode at all, resulting in the zero-current response. (**f**) Photonic wavelength shift of the resonance dip of an electro-optical surface functionalized with silane chemistry upon exposure to a thiolated MB probe. The black arrows represent the injection of the thiolated MB probe (blue overlay) and phosphate buffer (100 mM pH 7; orange overlay), respectively. Assuming a homogenous layer of a known refractive index, the wavelength shift can be employed to calculate the thickness of the MB layer. In our case, for a shift of 0.58 nm, a thickness of 2.6 nm±0.05 nm is obtained (for details, see Supplementary Fig. 14).

Starting with undoped silicon on insulator (SOI) material with a top waveguide layer of 220 nm thickness, we used thermal diffusion doping with a relatively low temperature (845 °C) to create a thin, highly doped silicon layer located at the substrate surface. Measurements of the doping profile reveal a doping density, *n*=10^18^−10^20^ cm^−3^ in the top 15 nm (Fig. 1c; see Supplementary Figs 1 and 2). This high doping density renders the silicon sufficiently conductive for use as an electrode in electrochemistry (Fig. 1e). Because the *n*-doped layer is so thin, the doping density averaged through the depth of the waveguide layer remains sufficiently low (*n*=7.5 × 10^16^ cm^−3^ over 220 nm) that optical losses are minimized. This is shown in Fig. 1d that compares otherwise identical ring resonators fabricated in *n*-type surface-doped and -undoped silicon (the correlation between doping density and Q-factor is shown in Supplementary Fig. 3, while an assessment of the Q-factor of a lossy cavity is given in the Supplementary Note 1). Critically, the Q-factor of the doped resonators is ∼50,000; typical of optical ring resonator sensors^7^,^8^,^12^,^13^ and is only marginally lower than that of the undoped device (65,000).

To explore the dual sensing capabilities of our device, we used the electrophotonic technology to monitor and quantify electrochemical reactions occurring at the silicon surface. Using established silane chemistry^14^ (see Supplementary Method 1 for details), the surface of the photonic electrode was modified to render it thiol-reactive, enabling conjugation of a redox-active methylene blue (MB) analogue labelled with a thiol linker^15^,^16^. Figure 1f demonstrates the spectral shift in photonic resonance that results after exposure of the functionalized surface to the thiolated MB. The wavelength shift (Δ*λ*=0.58 nm) is indicative of the formation of a surface layer. This resonant shift can be used to estimate the molecular density of the monolayer based on an assumption of the refractive index^17^. However, by also performing measurements in the electrochemical domain (Fig. 1e), it is not only possible to measure precisely the molecular density of the monolayer (without any assumptions about the optical properties of the layer) but also confirm its redox activity. The large oxidation and reduction current peaks observed in cyclic voltammograms of the MB-modified electrophotonic surface confirm the redox activity of the immobilized MB layer. Furthermore, from the area under the redox current peaks we can also precisely calculate the density of surface-attached MB molecules (2.2 × 10^12^ molecules cm^−2^, see Supplementary Fig. 4 and Supplementary Table 1). We note that this compares well with the value of molecular density of 1.5 × 10^12^ molecules cm^−2^ estimated by a simulation of the ring resonator in which we have assumed a layer refractive index of 1.5 (ref. 18; see Supplementary Fig. 5 and Supplementary Method 4).

### Photonic measurements of electrochemical reactions

**Figure 2:**
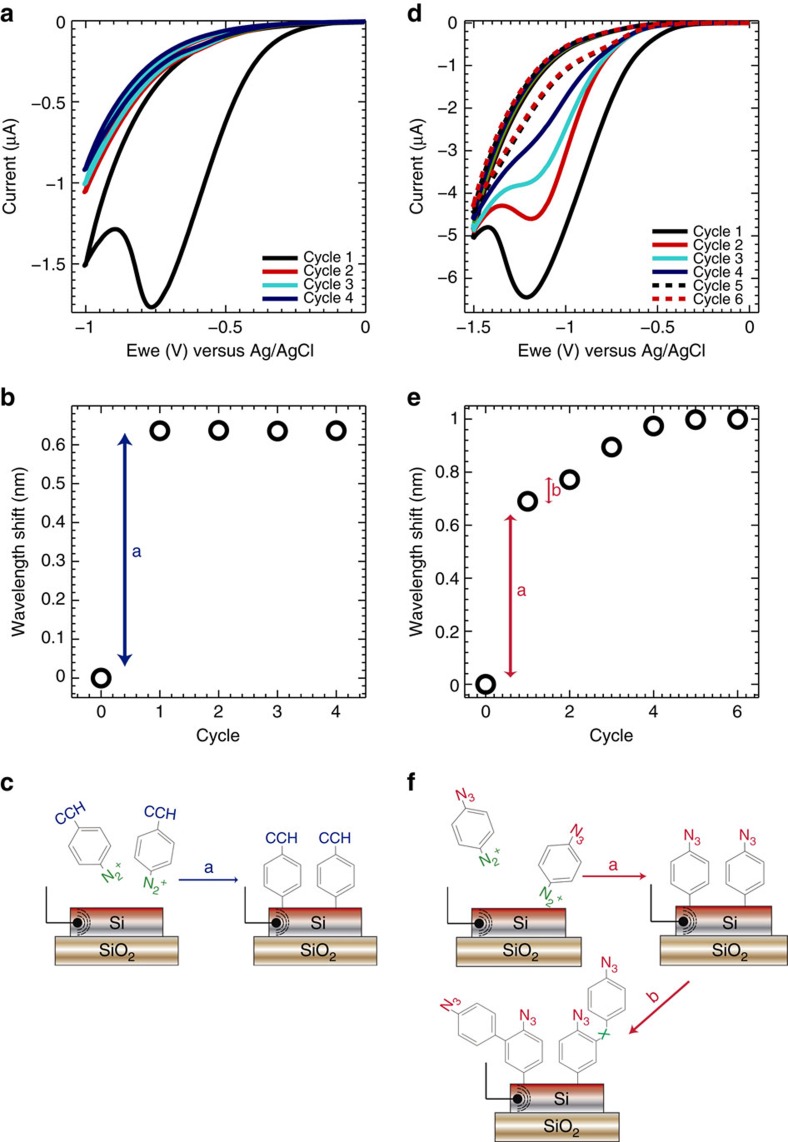
Surface alkyne and azide bio-functionalization of the electrophotonic biosensor. (**a**) Electrochemical response during diazotization and electrografting of 4-ethynylbenzene diazonium at 50 mV s^−1^. The reduction peak at −0.8 V corresponds to the cleavage of the dinitrogen in the 4-ethynylbenzene molecule. The fact that no reduction peaks are observed in subsequent cycles of the electrode potential suggests complete assembly of a molecular layer during the first voltage sweep. (**b**) The electroreduction is accompanied by a change in the refractive index around the waveguide (indicated by ‘a'), which leads to a 0.62 nm wavelength shift. (**c**) Sketch of the electrografting of the 4-ethynylbenzene molecule to the silicon photonics electrode. (**d**) In contrast, diazotization and electrografting of azidoaniline leads to multiple peaks in the reduction current that occur with each 50 mV s^−1^ cycle of the electrode potential. (**e**) The photonic response provides insight into this reaction, as wavelength shifts associated with each cycle are also observed (‘a' and ‘b' for the first and second cycles, respectively). Here the wavelength shift after the first reductive cycle (0.65 nm) is comparable to that observed for 4-ethynylbenzene diazonium, confirming the assembly of a well-packed monolayer after the first cycle (**d**,**e**). Subsequent cycles of the electrode voltage leads to the formation of a multilayer structure because of highly reactive radical chemistry. (**f**) Sketch of the electrografting of azidoaniline to the silicon photonics electrode. X indicates unspecified chemical functionality.

The combination of electrochemical and photonic sensing not only provides access to complementary information, but also the ability to regulate the local surface chemistry via electrochemical processes *in situ*. For example, (Fig. 2a) shows electrochemical modification of the photonic electrode surface via *in situ* diazotization and electrografting of 4-ethynylbenzene diazonium. The electrochemical reaction results in a prominent reduction current peak that is observed only for the first cycle of the electrode potential. This electrografting is accompanied by a shift in the refractive index of the ring resonator (Fig. 2b,c), consistent with the formation of a molecular layer (control measurements without diazonium in solution are shown in the Supplementary Fig. 6 and XPS confirmation of the surface chemistry are shown in Supplementary Figs 7–9). The relatively large optical shift suggests the formation of a densely packed molecular layer. This is confirmed from the magnitude of the electrochemical reduction current peak from which we calculate a molecular density for the electrografted layer of 3.7 × 10^13^ molecules cm^−2^ (see Supplementary Fig. 3). We are therefore able to form diazonium monolayers of equivalent density to those reported in the literature for other electrode materials^19^. The absence of reductive peaks or shifts in resonant frequency with continued cycling of the photonic electrode potential indicates that the surface reaction reaches completion after the first reductive cycle.

**Figure 3:**
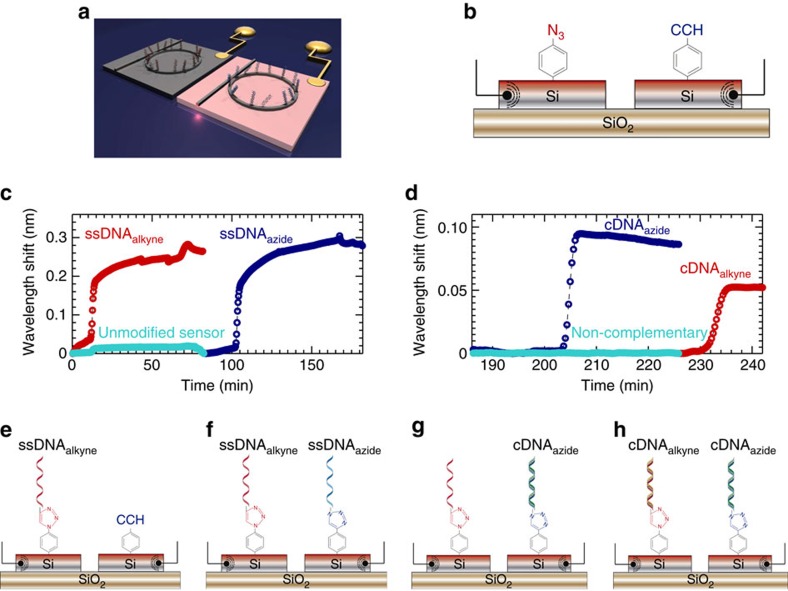
Selectively functionalized optical ring resonator DNA array. (**a**) Electrografting of diazonium ions is used as pattering method to selectively functionalize a pair of optical ring resonator sensors within the same microfluidic chamber. (**b**) Each sensor is electrically isolated; thus, a different diazonium salt can be electrografted on each sensor to provide an orthogonal chemical functionality. (**c**) The individual resonators are then functionalized with modified DNA sequences using copper-catalysed azide–alkyne click (CuAAC) reaction, showing selective binding to the microrings functionalized with the pertinent chemical moiety. No binding is observed using an unmodified microring, as illustrated by the turquoise trace. (**e**) First, the azide-modified sensor is functionalized with the ssDNA_alkyne_ sequence. (**f**) Second, the alkyne-modified sensor with the ssDNA_azide_ sequence. Both ssDNA_alkyne_ and ssDNA_azide_ were added at a 2 μM concentration. This immobilization process was performed using potassium phosphate buffer (100 mM, pH 7). (**d**) The selectivity of the functionalization process is confirmed by exposing the sensor array to DNA of the reverse complement of ssDNA_azide_ and ssDNA_alkyne_. We note that no hybridization is observed when challenging the sensor to non-complementary DNA, as illustrated by the turquoise trace. (**g**,**h**) Injection of the reverse complements of ssDNA_azide_ and ssDNA_alkyne_, labelled as cDNA_azide_ and cDNA_alkyne_, respectively. The reverse complements cDNA_azide_ and cDNA_alkyne_ were injected at 400 nM concentration in Tris-EDTA (TE) containing 1 M NaCl.

In contrast, when azidoaniline is used for *in situ* diazotization and electrografting of the photonic electrode (Fig. 2d,e), each of the first four voltammogram cycles contains reductive current peaks of decreasing amplitude (Fig. 2d). Each potential cycle also leads to a corresponding shift in the resonance frequency of the microring photonic sensor (Fig. 2e). This confirms that the electroreduction processes measured in cycles 1–4 result from modification of the electrode surface, rather than from an electrochemical reaction of excess diazonium ions in the solution phase. The magnitude of the reduction peak following the first potential cycle corresponds to a packing density of 2.1 × 10^14^ molecules cm^−2^ in the electrografted layer. This high surface density of molecules and the large frequency shift indicate the formation of a complete monolayer at the silicon surface. The combination of electrochemical and photonic measurements thus enables us to deduce that the subsequent electroreduction processes occurring in cycles 2–4 correspond to the formation of a multilayer structure at the electrode. Multilayers are often observed in diazonium electrografting^20^, and the mechanism of formation involves highly reactive aryl radicals that are generated during diazonium electrografting (Fig. 3f). Our observation that multilayer formation occurs for the phenylazide diazonium electrografting reaction but not for the phenylacetylene diazonium process is consistent with previous studies, which have shown that the propensity for multilayer formation is highly dependent on the structure of the diazonium (see Supplementary Method 2 for details about the synthesis of the diazonium salts)^21^.

### Site-selective functionalization

Critically, the electrografting reaction is spatially localized and occurs only at the electrode^22^. We have exploited this site-selective control of surface chemistry to develop a multiplexed photonic sensor array in which each microring within the array is functionalized selectively with a different ‘probe' molecule. A bi-functionalized sensor was constructed by modifying the surfaces of two, individually addressable photonic electrodes, fabricated within the same device via electrografting of ethynylaniline on one electrode, and azidoaniline on the second (Fig. 3a,b). We subsequently exploit the differences in chemical functionality of the two ring resonators for site-specific DNA bioconjugation (DNA sequences given in Supplementary Table 2). The copper-catalysed azide–alkyne click reaction simply and specifically binds alkyne (RC≡CH) surface moieties to azide (R′N=N^+^=N^−^) groups on a biomolecule and *vice versa*. This reaction can either be electrically controlled via *in situ* electroreduction of Cu^2+^ to Cu^1+^ (ref. 23), or using a solution-based reducing agent, as described in the Supplementary Method 3. Thus, single-stranded DNA containing an azide group, ssDNA_azide_, can only react with the ring resonator functionalized with alkyne groups, and single-stranded DNA containing an alkyne moiety, ssDNA_alkyne_, can only react with the azide-modified ring resonator. Conjugation of DNA to the modified resonator surfaces was monitored optically and in real time, as shown in Fig. 2c. The saturation in wavelength shift indicates the copper-catalysed azide–alkyne click reaction proceeds to completion for both ssDNA_azide_ and ssDNA_alkyne_, while the magnitude of the shift in wavelength is comparable for both resonators, indicating a similar density of the two DNA monolayers (confirmed via Quartz Crystal Microbalance with Dissipation Monitoring as described in Supplementary Figs 10 and 11).

Finally, multiplexed photonic sensing was demonstrated by adding single-stranded DNA complementary to ssDNA_azide_ to the two photonic electrode device. A corresponding shift in resonance is observed only on the microring functionalized with ssDNA_azide_, attributable to the formation of a double-stranded DNA complex (Fig. 3d). The lack of resonance shift on the microring derivatized with ssDNA_alkyne_ not only highlights the high spatial selectivity of the electrografting process but also confirms the multiplexing capability of the sensor. Similarly, upon exposure to 400 nM DNA complementary to ssDNA_alkyne_, we observe a shift in resonance only on the optical ring functionalized with ssDNA_alkyne_ (Fig. 3d).

## Discussion

We have created a device capable of multiplexed detection that consists of a pair of photonic electrode ring resonators in the same microfluidic chamber. Site-selective modification of the sensor surface was achieved using the electrochemical grafting of aryl diazonium salts, a highly versatile method for functionalization of electrode surfaces^24^. The wide range of diazonium ions, which are available, or can be generated *in situ* from amine molecules, can be exploited to introduce reactive groups on the surface of silicon, which will crosslink to complementary chemical moieties on a very large number of (bio)molecules. For example, a further example of *in situ* diazotization is shown in the Supplementary Figs 12 and 13 where the photonic electrode is modified with thiol-reactive maleimide groups. A prominent feature of electrochemical grafting as an approach for surface functionalization is that the reaction is highly localized at the electrode surface. This means that the grafting of organic materials can be controlled at the micrometre scale (the scale of individual microrings), and possibly even on submicrometre scales. This spatial resolution is orders of magnitude higher than that achieved using conventional ink-jet^25^ and ink-dot printing approaches. This capability is fundamental for the design of very high densities of biosensor arrays; for example, one can envisage a sensor with a total area of only a few square micrometres, yet with multiple different sensing sites, such that multiplexed sensing of panels of biomarkers or multimodal sensing inside eukaryotic cell becomes a realistic possibility. Our technology also offers the ability to tailor and optimize the light–matter interaction through control of the geometry of the photonic sensor, something that cannot be done using other combined electrochemical–optical sensors such as electrochemical surface plasmon resonance sensors^26^.

## Methods

### Device fabrication

All devices consist of an optical ring resonator structure etched into the *n*-type doped silicon layer of a SOI wafer (SOITEC, France). The wafer consists of a 220 nm-thick layer of silicon on top of a 2 μm layer of buried oxide. Doping of the 220 nm silicon layer was achieved with a thermal diffusion method to drive the donors from a solid source (Phosphorus Grade PH-950, Saint-Gobain Ceramics, USA) into the silicon layer. We employed a furnace oven with a nitrogen environment, ramping the temperature up to 845 °C at 5 °C min^−1^, where it was maintained for 10 min. Once doped, electrical contacts are formed on the SOI substrate by means of thermal evaporation of 200 nm of aluminium, followed by a protective layer of 20 nm of nickel and 200 nm of gold (nickel is required to avoid the growth of further insulating intermetallic layers between aluminium and gold and to improve gold adhesion). The ring resonator structure (we chose the all-pass configuration as it offers higher Q-factors, hence improving the sensitivity of the device) is patterned by electron beam lithography (acceleration voltage of 50 kV with a step size of 4 nm) using the positive e-beam resist AR-P 6200.09 (Allresist, Germany) at a thickness of 350 nm. Ring resonators are patterned with waveguide dimensions of 500 × 220 nm and a radius of 30 μm. The pattern was transferred into the silicon layer by dry etching employing a reactive ion etcher (gas composition of SF_6:_CHF_3_ 1:1.16). The silicon was shallow-etched to ensure ∼30 nm of silicon between the access waveguide and the optical cavity remained in order to ensure electrical continuity between the ring cavity and the electrical contact placed at the edge of the device. Finally, the silicon layer was piranha-cleaned (H_2_SO_4_:H_2_O_2_ mixture at a 7:3 ratio) and oxygen plasma-activated to bind a polydimethylsiloxane (184 Sylgard) microfluidic channel to the device.

### DNA sensor array fabrication

Our DNA sensor array is made of two identical optical ring resonators separated by 250 μm. The devices have the same physical dimensions and are fabricated using the method discussed above. Two contact pads are placed at the edges of the sample to electrically address each ring resonator individually. To achieve the desired selective functionalization, both sensors have to be electrically isolated. We achieve this by etching a trench between both sensors, hence electrically isolating the two ring cavities. Etching is performed by dry etching a 100 μm-wide trench patterned using a 300 nm-thick layer of S1818 photoresist (MICROPOSIT, DOW chemical company, USA) through ultraviolet exposure. Once the conductive layer of silicon between the devices is removed, a polydimethylsiloxane microchannel is bound as described above.

### Electrochemical/optical set-up

Our experiments were carried out in a modified endfire transmission set-up, where electrochemical functionalities were incorporated. Light from a single-mode fibrecoupled to a broadband amplified spontaneous emission source (1,520–1,620 nm) was collected and collimated by an aspheric lens and splitter cube to a × 60 lens. The spot from this lens was focused on an access waveguide on the edge facet of the fabricated SOI chip. The required alignment was performed using an infrared camera with a × 20 objective while illuminating the sample with a white light source. Light propagating through the sample to the back facet was then collected and collimated with a × 40 lens, and sent through free space to a focusing aspheric lens on the facet of a single-mode fibre. This light was finally split using a splitter fibre to an optical spectrum analyser. Continuous measurements of the transmission spectrum were taken with the optical spectrum analyser, where the resonance wavelength is continuously Lorentzian-fitted and monitored. A syringe pump and fluidic valve were used to ensure a controlled delivery of samples through the inlet and outlet ports of the microfluidic channel. The silicon substrate was used as the electrochemical ‘working' electrode and, via a potentiostat, the potential of the silicon electrode was controlled relative to that of an Ag/AgCl double-junction reference electrode, while a platinum ‘counter', electrode completed the circuit.

### Data availability

All data created during this research will be available online from the University of York Data Catalogue at DOI: 10.15124/419c1760-fd74-4046-ba90-fb53710430f3.

## Additional information

**How to cite this article:** Juan-Colás, J. *et al*. The electrophotonic silicon biosensor. *Nat. Commun.* 7:12769 doi: 10.1038/ncomms12769 (2016).

## Supplementary Material

Supplementary Information Supplementary Figures 1-14. Supplementary Tables 1-2, Supplementary Note 1, Supplementary Methods 1-4 and Supplementary References
