# Supplementary material for: The electrophotonic silicon biosensor
Source: Nat Commun. 2016 Sep 14;7:12769. doi: 10.1038/ncomms12769 (PMC5027286; doi:10.1038/ncomms12769)
Supplement: Supplementary Information — Supplementary Figures 1-14. Supplementary Tables 1-2, Supplementary Note 1, Supplementary Methods 1-4 and Supplementary References [file ncomms12769-s1.pdf]

The electro-photonic silicon biosensor

Supplementary Information

José Juan Colás[1, 2]*, Alison Parkin[3*], Katherine E. Dunn[1], Mark G. Scullion[2], Thomas F. Krauss[2] and Steven D. Johnson[1]

[1] The Department of Electronics, University of York, Heslington, York, YO10 5DD, UK

[2] Department of Physics, University of York, Heslington, York, YO10 5DD, UK

[3] Department of Chemistry, University of York, Heslington, York, YO10 5DD, UK

*Correspondence: (J.J.C) jjc525@york.ac.uk, (A.P.) alison.parkin@york.ac.uk

Supplementary Figures

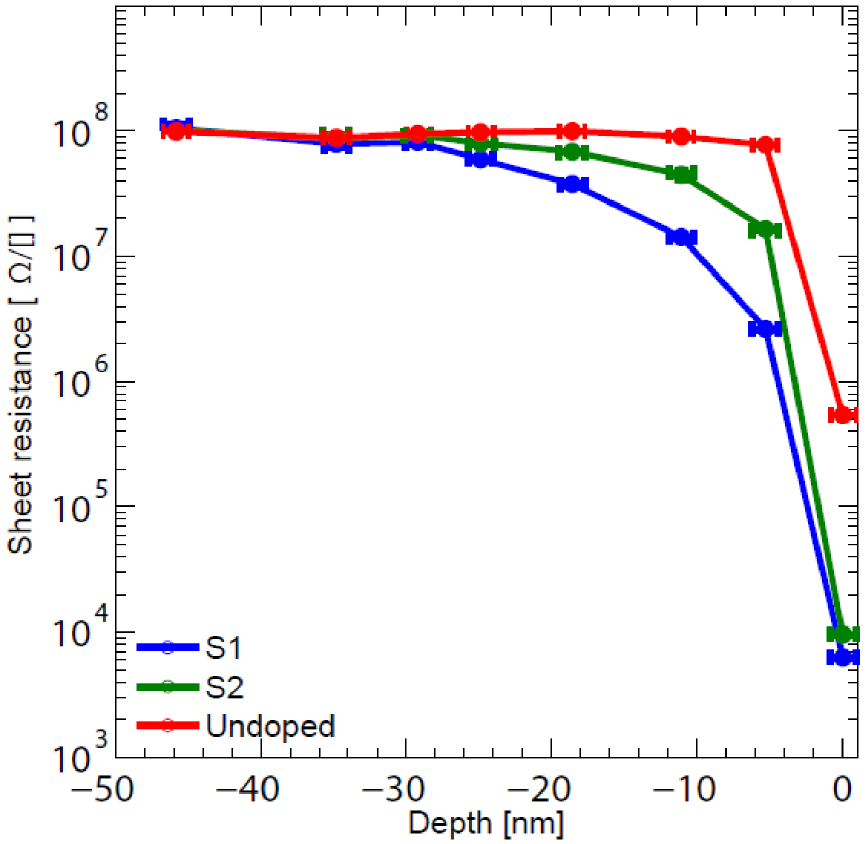

Supplementary Figure 1: Sheet resistance of three different samples, Undoped, S1 (7.5 x 10^{16} cm^{-3}) and S2 (5.2 x 10^{16} cm^{-3}), at different depths within the silicon layer. The intrinsic doping profile of the undoped silicon sample is shown in red, whereas the n-type doping of two samples, S1 and S2, is shown in blue and green. To measure the sheet resistance, a four-point probe setup was employed and the thickness measurements were performed using an in-house developed ellipsometer. The error bars represent the systematic error in the depth measurement performed by ellipsometry.

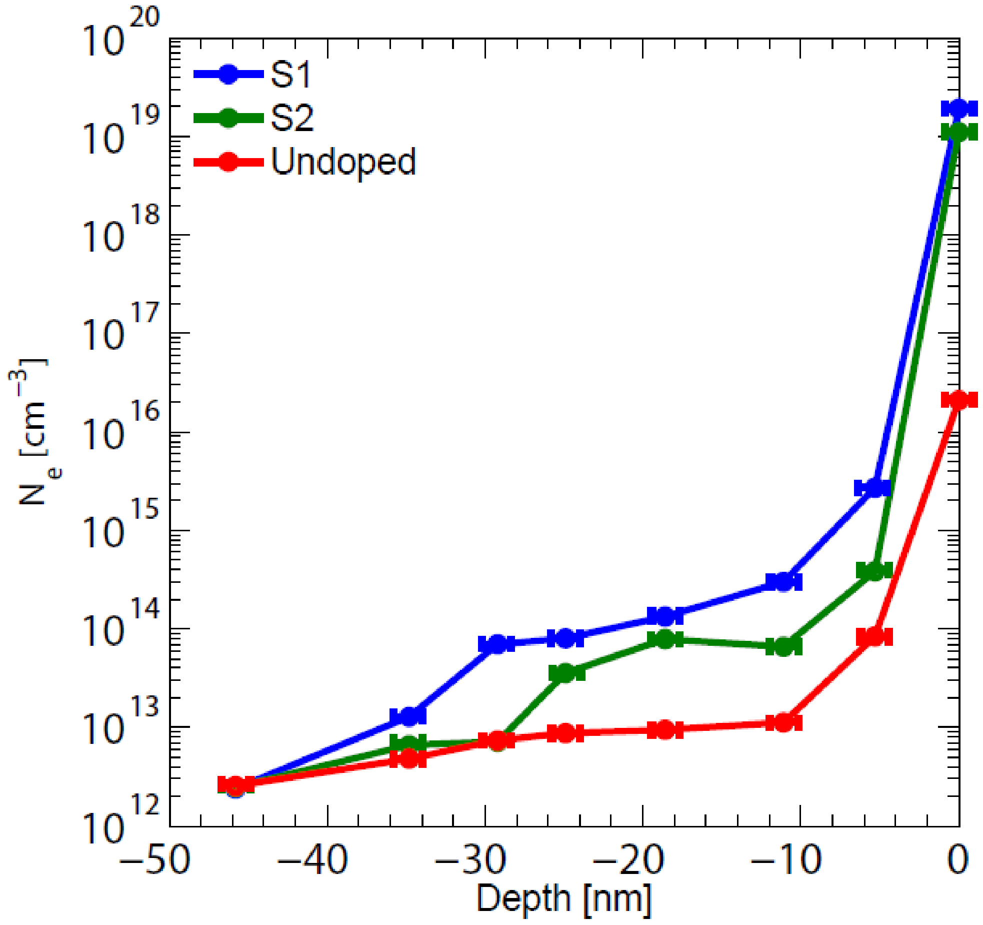

Supplementary Figure 2: Estimated doping concentration of three different samples, Undoped, S1 (7.5 x 10^{16} cm^{-3}) and S2 (5.2 x 10^{16} cm^{-3}), as a function of etch depth where zero on the x-axis corresponds to the silicon surface. The gradient of the doped silicon layer was modelled as a number of resistances in parallel where each represents the resistivity of a layer. Transferring these values to an equivalent electrical circuit it is possible to deduce the resistivity of each layer. Subsequently, the doping density of each layer is obtained numerically by evaluating the resistivity value in the mobility model of Klaaseen [1] [2]. A highly doped thin film is obtained in the top 15 nm of the silicon layer following our diffusion technique discussed in the methods section "device fabrication". The error bars represent the systematic error in the depth measurement performed by ellipsometry.

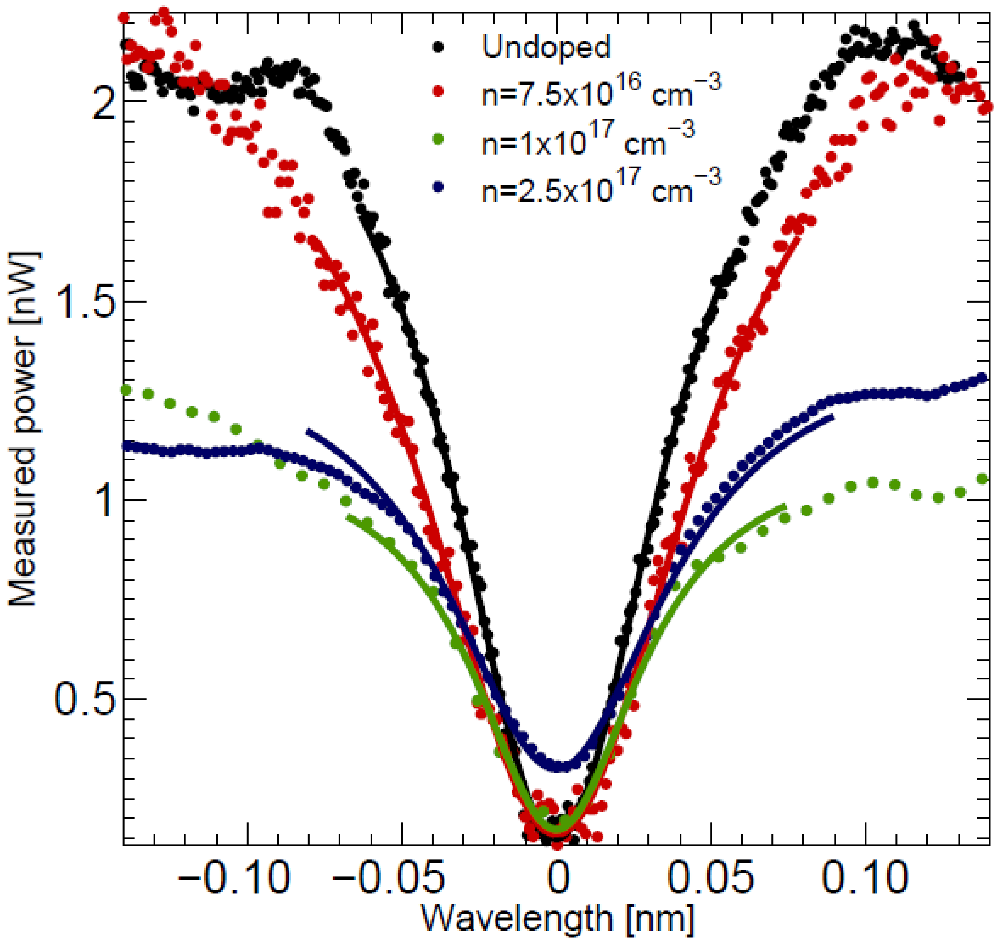

Supplementary Figure 3: Dependence of the Q-factor and the extinction ratio on the average doping density of the material. The effect of the doping concentration against the spectral performance of the device is twofold, as both Q-factor and dynamic range of the cavity are decreased. This is attributed to the increase in the optical loss due doping of the substrate. Q-factors of 45,000 and 30,000 were measured for doping densities of 1×10^{17} cm^{-3} and 3×10^{17} cm^{-3}, respectively; while a Q-factor of 50,000 is obtained for $7.5\text{x}10^{16}$ cm^{-3}. For our working doping density ($n = 7.5\times10^{16}$ cm^{-3}), the broadening of the resonance dip is minimal. However, for higher doping densities, even though the Q-factor does not decrease dramatically, the extinction ratio is noticeably reduced.

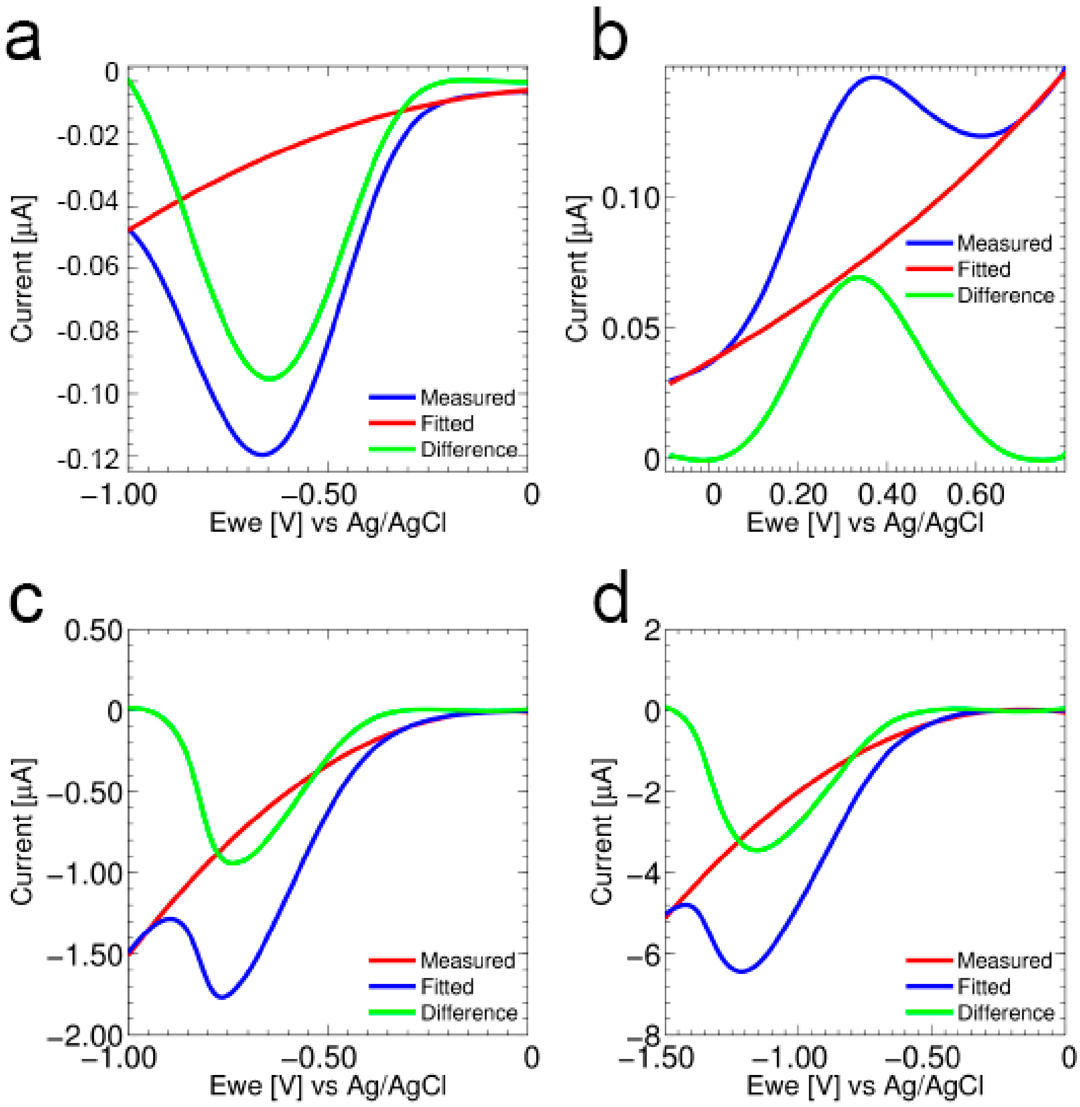

Supplementary Figure 4: Surface coverage estimation analysis. (a) Surface coverage estimation analysis of the experiment shown in Fig 1e for a doping density of $n = 7.5 \times 10^{16}$ cm^{-3}. (b) Surface coverage analysis of the methylene blue probe immobilized in Supplementary Figure 13b. (c) Surface coverage analysis of the electroreduction of the 4-ethynylbenzene diazonium salt shown in Fig. 2a. (d) Surface coverage analysis of the electroreduction of the azidoaniline shown in Fig. 2d.

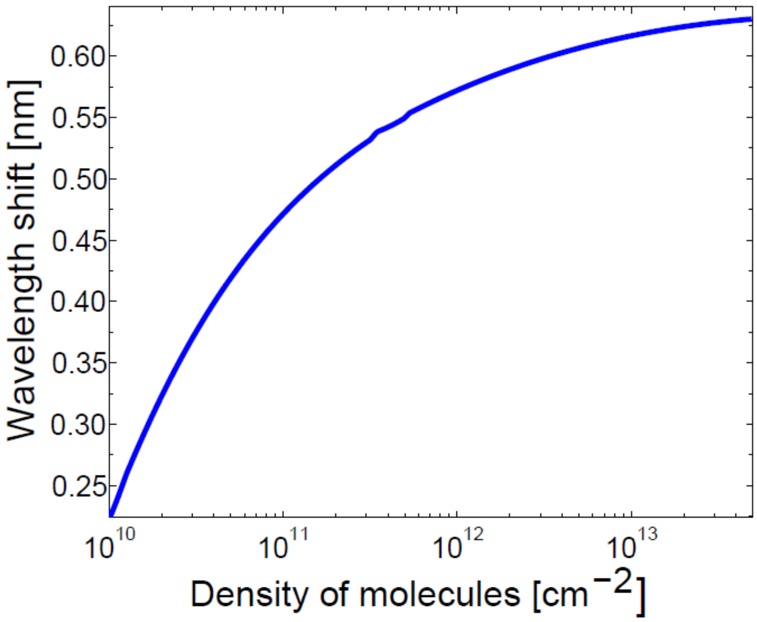

Supplementary Figure 5: Wavelength shift of our ring resonator sensor against the surface coverage of the MB molecule of Fig. 1f. The simulation method is described in the Supplementary Method 4.

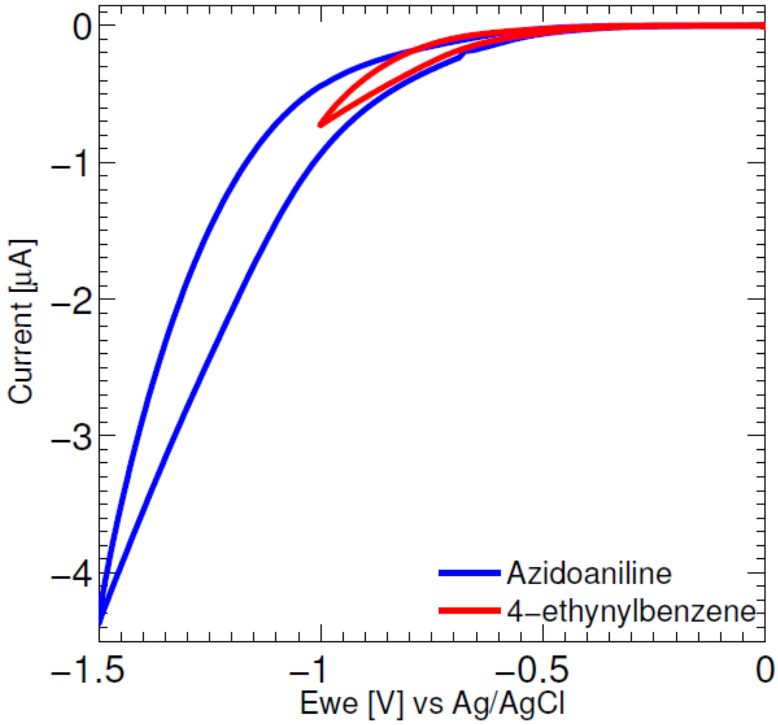

Supplementary Figure 6: Control experiments of the electroreduction of both azidoaniline and 4-ethynylbenzene diazonium solutions without adding the electroactive active molecule. As expected, no reduction peaks are observed in any of the voltammograms because no electroactive molecule is present in the solution. All other chemicals, as discussed in the Supplementary Method 2, were added to the mixture. Cyclic voltammograms were measured at 50 mV s^{-1}.

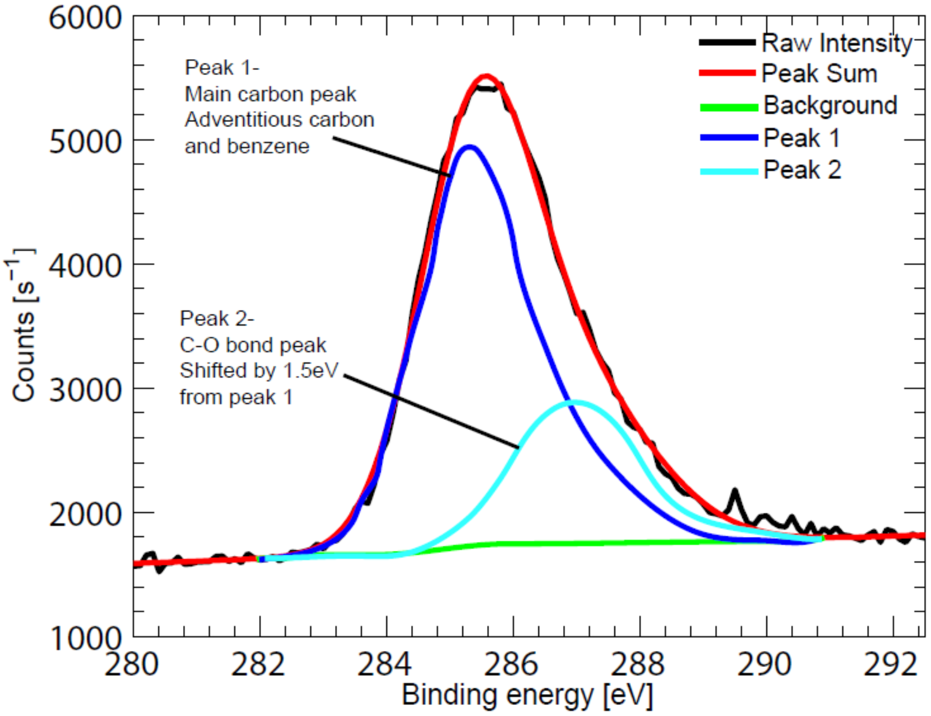

Supplementary Figure 7: X-ray photoelectron spectroscopy (XPS) spectrum of a silicon substrate without biomodification. The main carbon contamination peak is observed at 285 eV.

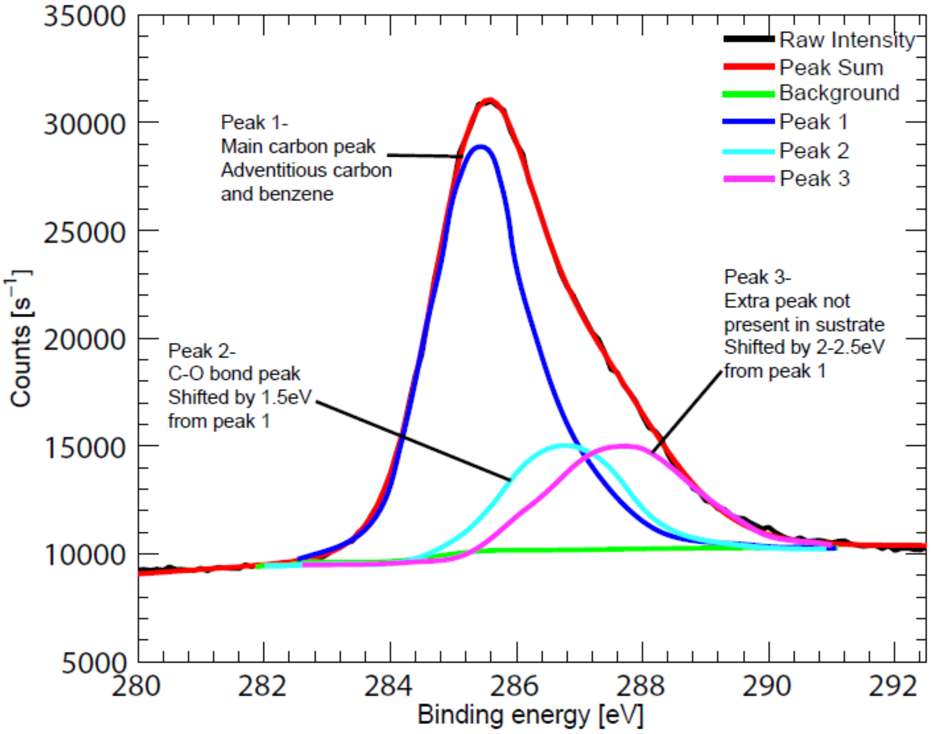

Supplementary Figure 8: XPS spectrum of a silicon substrate where the diazonium salt of aniline has been previously electrografted. We observe a new peak, shifted 2 – 2.5 eV from the main carbon peak due to the presence of electrografted diazonium molecule.

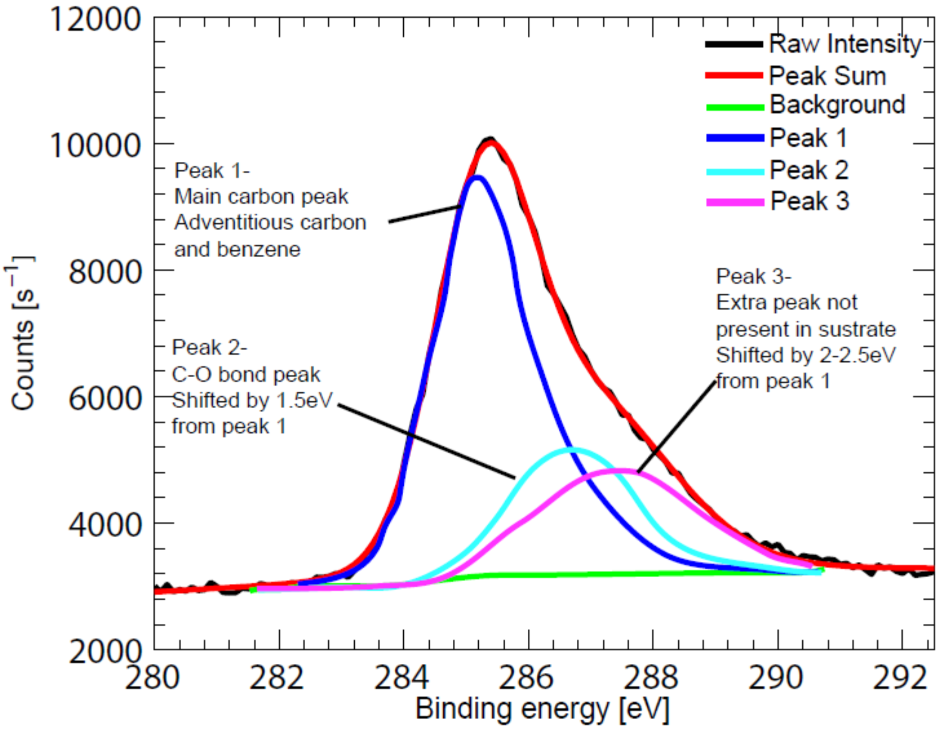

Supplementary Figure 9: XPS spectrum of a silicon substrate where the diazonium salt of azidoaniline has been previously electrografted. As in Supplementary Figure 8, we observe a new peak, shifted 2 – 2.5 eV from the main carbon peak due to the presence of electrografted diazonium molecule.

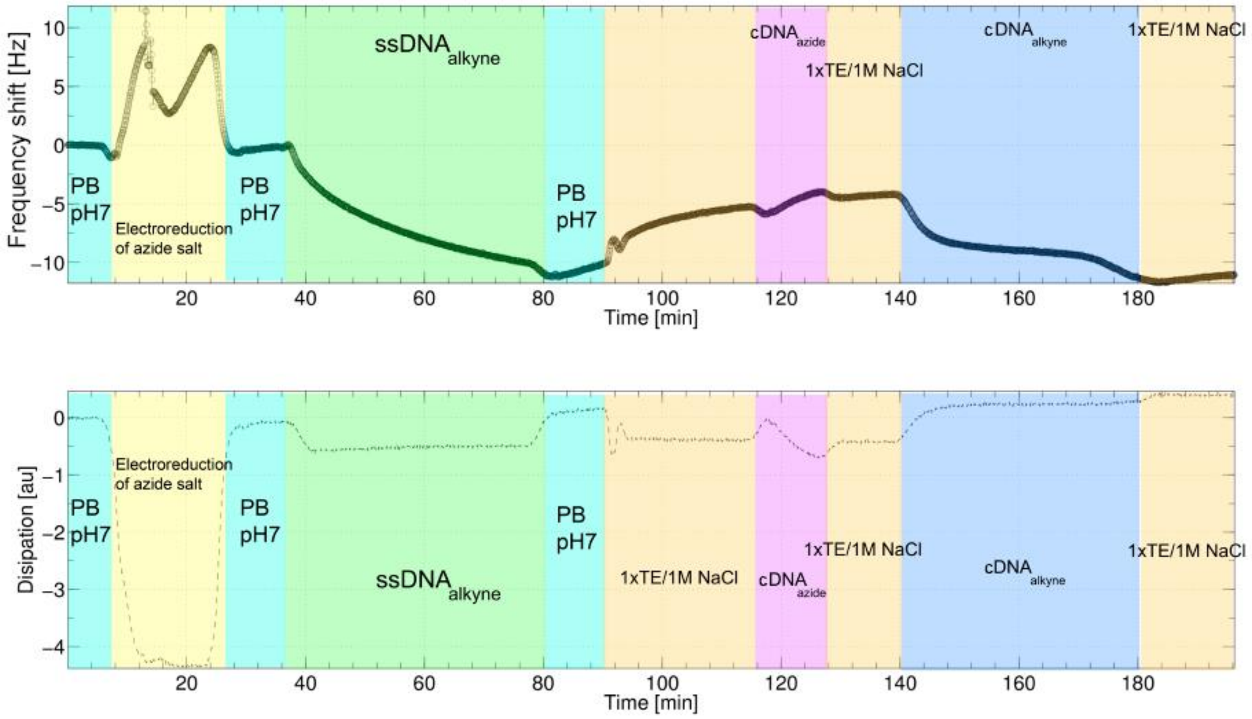

Supplementary Figure 10: Azide modified surface control experiment for selective DNA hybridization. Control experiments to analyse the surface modification chemistry were performed on a Quartz Crystal Microbalance with Dissipation Monitoring (QCM-D) instrument (Q-Sense E4). Gold electrodes were chosen because they were compatible with all the reactions involved. An electrochemical module (QEM 401) was fitted to the instrument in order to study and optimize the electroreduction chemistry. A constant flow rate of 20 µL min^{-1} was kept throughout the measurements. Firstly, the QCM-D gold sensor was modified through diazotization and electrografting of azidoaniline. $ssDNA_{alkyne}$ (1 µM) was then injected and immobilized to the surface through azide-alkyne Huisgen cycloaddition as discussed in the Supplementary Method 3. No change in mass or dissipation was observed following injection of non-complementary DNA_{azide} (400 nM), indicating no hybridization with the strand fixed to the surface. In contrast, hybridization is observed when flowing the complementary $cDNA_{alkyne}$ (400 nM) through the sensor, hence demonstrating selective DNA sensing. 1xTris-EDTA (1xTE) buffer with 1M NaCl was used during the hybridisation assay.

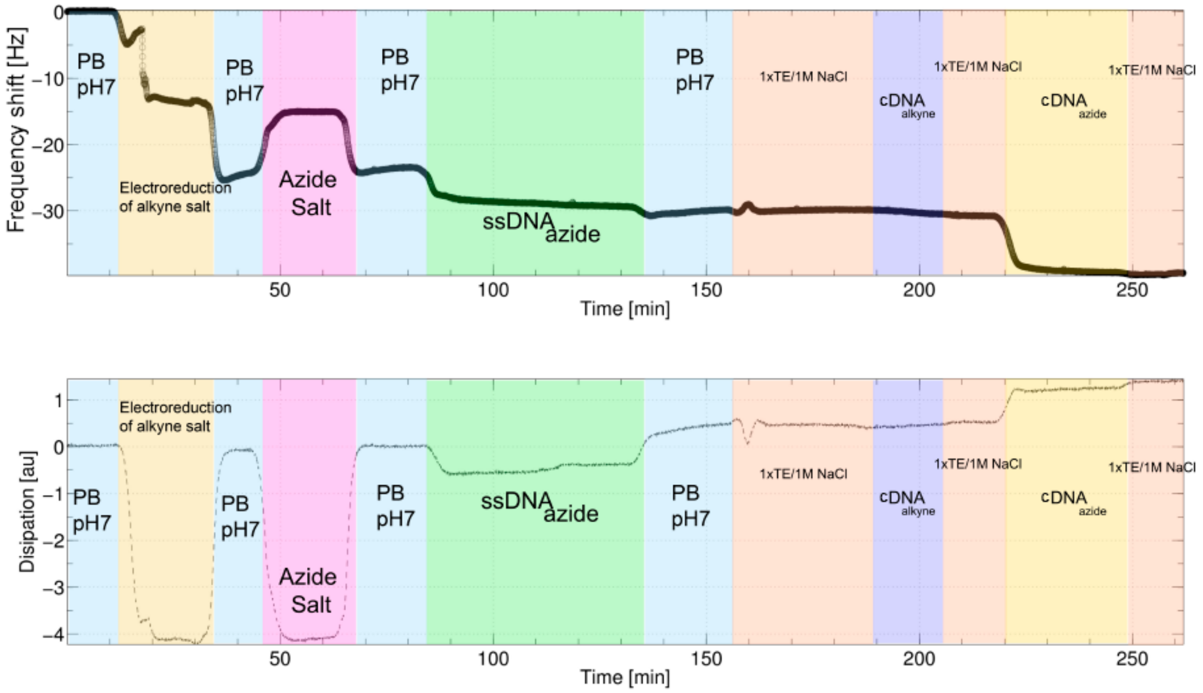

Supplementary Figure 11: Alkyne modified surface control experiment for selective DNA hybridization. Firstly, the QCM-D gold sensor was modified through diazotization and electrografting of 4-ethynylbenzene diazonium. The modified surface was next exposed to the solution required for azide modification, as required during bi-functionalisation of the photonic array. $ssDNA_{azide}$ (1 µM) was then injected and immobilized to the surface through azide-alkyne Huisgen cycloaddition as discussed in the Supplementary Method 3. The increase in mass is consistent with the assembly of a $ssDNA_{azide}$ layer and suggests no deterioration of the chemical activity of the surface alkyne after exposure to the chemicals required for electroreduction of azidoaniline. No change in mass or dissipation was observed following injection of non-complementary DNA_{alkyne} (400 nM), indicating no hybridization with the strand fixed to the surface. In contrast, hybridization is observed when flowing the complementary $cDNA_{azide}$ (400 nM) through the sensor, hence demonstrating selective DNA sensing.

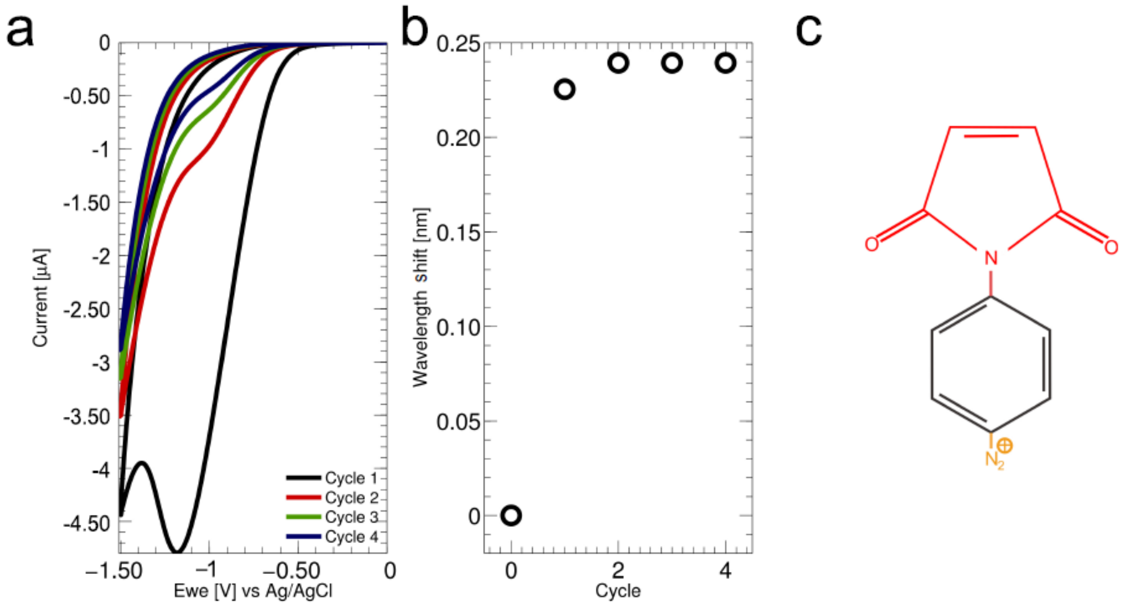

Supplementary Figure 12: Maleimide modification of the electro-optical sensor. (a) Electroreduction of the diazonium salt of n-(4-aminophenyl)maleimide on the electro-optical sensor. The single current peak observed in the cyclic voltammogram indicates an irreversible electrochemical reduction reaction, indicative of electrografting with diazonium. Voltammograms were performed at 50 mV s^{-1}. (b) Optical shift induced by the grafting of the maleimide molecule. (c) Structure of the diazonium salt synthesized from n-(4-aminophenyl)maleimide.

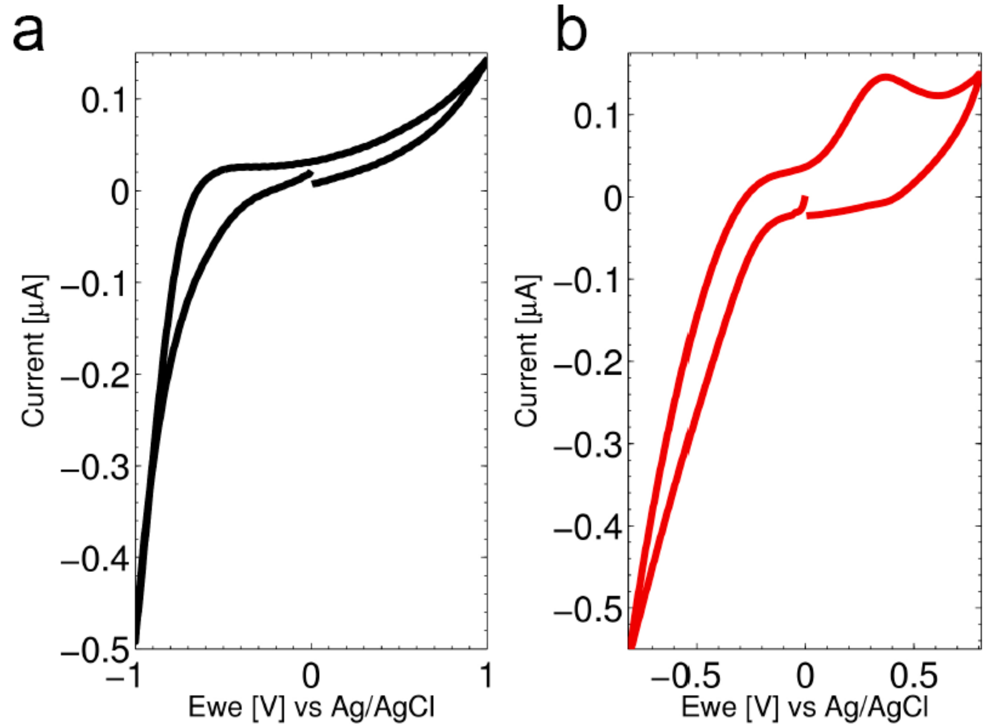

Supplementary Figure 13: Immobilisation of the redox-active methylene blue (MB) probe labelled with a thiol-linker on the maleimide modified silicon substrate. (a) Cyclic voltammogram of the maleimide modified silicon substrate at 50 mV s^{-1} using a 100 mM potassium phosphate pH 7 electrolyte. No electrochemical activity is displayed after the maleimide functionalization of the surface. (b) Cyclic voltammogram of a methylene blue functionalized surface which had previously been functionalised with maleimide. The MB probe was incubated for 60 min at concentration of 0.75 mM in water. An oxidation peak is observed at a voltage value of 0.35 V indicating the oxidation of the MB probe. No reduction peak can be seen as the background current is higher than that induced by the reduction of the molecule. From the area under the oxidation peak we calculate a surface density 2.56×10^{12} molecules cm^{-2} is achieved, similar to that reported on gold electrodes [3].

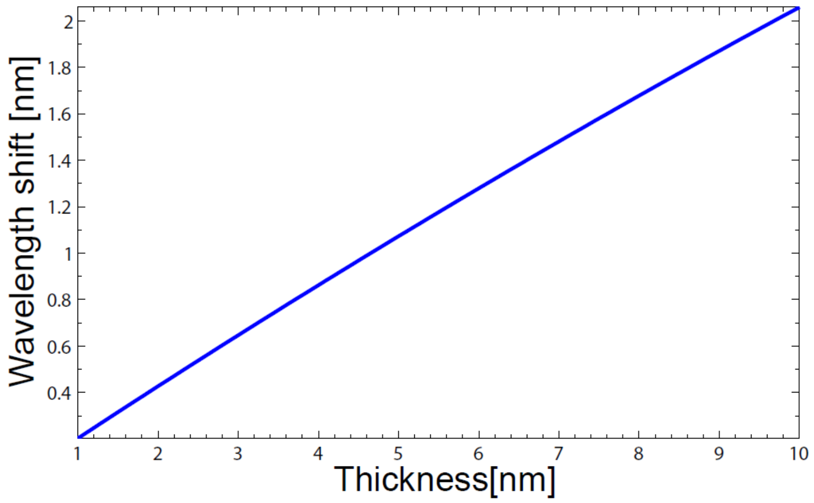

Supplementary Figure 14: Thickness estimation of a MB monolayer. As in [4], the resonance shift was simulated assuming a homogenous layer of a fixed refractive index but with variable thickness. Here the refractive index of the layer was fixed to 1.5 [5], and the refractive index of the buffer surrounding the sensor was 1.312. Simulations were performed using COMSOL multiphysics. The thickness of the MB layer is obtained at the intersection of the curve with the measured value of the optical shift. In our case, for a shift of 0.58 nm, a thickness of 2.6 nm -/+ 0.05 nm is obtained.

Supplementary Tables

Supplementary Table 1: Surface coverage estimation for the redox active molecules discussed in Fig. 1e, Supplementary Figure 13b, Figure 2a and 2d. Firstly, the background, capacitive charging current is removed from the cyclic voltammograms by fitting a curve to those regions of the voltammogram where no redox activity is shown, which is then subtracted from the measured curve. After the background current has been subtracted, the peak current is integrated to get the transferred charge Q. This is then divided by the electrode area (known from the dimensions of the microfluidic channel) to obtain the relative charge per area in C cm^{-2}. Finally, this charge density is divided by e to obtain the number of electrons involved in the reaction and thus the number of molecules (the diazonium electrografting reaction is a single electron process, i.e. one electron per molecule, whereas the reversible redox reaction of methylene blue is a two electron process).

**Molecule/process**	**Transferred charge [C]**	**Number of surface molecules [molecules cm^{-2}]**
Thiolated-MB on thiol-modified silicon using silane chemistry (fig 1e)	1.13 x 10^{-8}	2.2 x 10^{12}
Thiolated-MB on maleimide-modified silicon using phenylmaleimide diazonium (Supp. Fig. 13b)	6.67 x 10^{-8}	2.56 x 10^{12}
Electrografting of 4-ethynylbenzene diazonium on silicon (Fig. 2a)	9.64 x 10^{-7}	3.7 × 10^{13}
Electrografting of azidoaniline diazonium on silicon (Fig. 2d)	5.47 x 10^{-6}	2.1 × 10^{14}

Supplementary Table 2: Designed DNA oligonucleotides sequences for the experiments reported in the results section "Selective functionalisation”. Single-stranded DNA oligonucleotides were acquired from Integrated DNA Technologies (IDT). The sequence with an azide modification and its reverse complement were randomly generated and then checked for secondary structure and unwanted interactions using NUPACK [6] (see http://www.nupack.org/). The strands with an alkyne modification and its reverse complement are designed to have no secondary structure and to be orthogonal to both the azide-modified strand and its reverse complement. The melting temperature (calculated using NUPACK considering the buffer 1xTE with 1 M NaCl) for the azide duplex is around 70 ºC, and 55 ºC for the alkyne duplex.

**Label**	**Sequence**	**Notes**
$ssDNA_{azide}$	5'-ACACGCATACACCCAT(3AzideN)-3'	Azide modified DNA
$cDNA_{azide}$	5'-ATGGGTGTATGCGTGT-3'	Reverse complement of the azide modified DNA
$ssDNA_{alkyne}$	5'-GTCATTTCTCTAAGTA(octadiynyl)-3'	Alkyne modified DNA
$cDNA_{alkyne}$	5'-TACTTAGAGAAATGAC-3'	Reverse complement of the alkyne modified DNA

Supplementary Notes

Supplementary Note 1: Assisted optical validation of the Q-factor of a lossy cavity

The effect of the doping on the optical performance of the devices was assessed analytically. By definition, the Q-factor of a cavity is the ratio between the energy stored in the cavity divided by the energy lost per cycle, multiplied by 2π. This can also be formulated as described in Supplementary Equation 1.

$$Q = 2\pi \times \frac{Energystored}{Energy\ lost/cycle} = 2\pi \times \frac{U_0}{U_0(1-e^{-\alpha L})} \quad (1)$$

In Supplementary Equation 1, α is the optical absorption at the working wavelength and L the length required to complete a single optical cycle. L can be rewritten as λ/n_{eff}, such that Supplementary Equation 1 is reformulated as:

$$Q = \frac{2\pi}{\left(1-e^{-\alpha\frac{\lambda}{n_{eff}}}\right)} \approx \frac{2\pi}{\alpha\frac{\lambda}{n_{eff}}} \quad (2)$$

$$(Using e^{x} = 1 + x, for\ \ small\ \ x)$$

The optical absorption, α, is obtained numerically using the method described in [7]. For a uniform doping concentration of $n_e \approx 7.5 \times 10^{16}$ cm^{-3}, we obtain an α of 4.2×10^{-5} µm^{-1}. Assuming n_{eff} = 2.35 (simulated through COMSOL Multiphysics) and λ=1580 nm, $Q_{abs}$is approximately 222,000.

From Fig. 1d, we obtain $Q_{doped} \approx 50{,}000$ and $Q_{undoped} \approx 65{,}000$. Since Q_{doped} can be obtained as: $\frac{1}{Q_{doped}} = \frac{1}{Q_{undoped}} + \frac{1}{Q_{\text{abs measured}}}$, the empirical $Q_{abs\ measured}$ is equal to:

$$\frac{1}{Q_{\text{abs measured}}} = \frac{1}{Q_{doped}} - \frac{1}{Q_{undoped}} = \frac{1}{50{,}000} - \frac{1}{65{,}000} \quad (3)$$

$$Q_{abs\ measured} = 216{,}000$$

This is in very good agreement with the estimate of 222,000 obtained by considering the loss induced by doping (Supplementary Figure 3).

Supplementary Methods

Supplementary Method 1: Silane chemistry to attach methylene blue to silicon

We followed the method described in [8] to attach the methylene blue redox molecule to the surface of our sensor. Briefly, a thiol modification at the surface was obtained by flowing a dilute solution of (3-Mercaptopropyl)trimethoxysilane (MPTS) (Sigma-Aldrich) in ethanol (400 µL of the MPTS in 10 mL of ethanol) through the PDMS channel. Once the solution completely filled the microfluidic channel, the flow was stopped and the reaction was left overnight. The sensor was then rinsed thoroughly with ethanol to remove any unreacted compound from the surface. Next, a 15 mM aqueous solution of Cu^{2+} ions was injected and left to incubate for 15 min. The modified surface was again rinsed, this time with potassium phosphate buffer (100 mM pH 7). Finally, the methylene blue molecule synthesized in [9] was incubated on the surfaced for approximately 60 min to achieve a high surface coverage.

Supplementary Method 2: Synthesis of the diazonium salts

We employed the diazonium salts of the molecules 4-ethynylaniline (Sigma-Aldrich), 4-azidoaniline (Sigma-Aldrich) and n-(4-aminophenyl)maleimide (Tokyo Chemical Industry UK). The preparation method comprises the treatment of the aromatic compounds with nitrous acid. The nitrous acid was generated *in situ* from a mixture of hydrochloric acid (HCl, Fisher Scientific) and sodium nitrite ($NaNO_2$, Fisher Scientific). To prepare the diazonium salt of 4-ethynylaniline, 35 mg was dissolved in 7 mL of 500 mM HCl at room temperature. Then 900 µL of 1 M aqueous $NaNO_2$ was slowly added to the mixture and left for 10 min with continuously stirring. The product was then injected into the PDMS channel and electroreduced. For the generation of diazonium salts of 4-azidoaniline or n-(4-aminophenyl)maleimide, 7 mL of 500 mM HCl was added to 70 µL of 100 mM 4-azidoaniline in methanol or 200 mM n-(4-aminophenyl)maleimide in acetonitrile, respectively. Finally, 280 µL of 100 mM $NaNO_2$ was added and stirred for 15 min before electroreducing.

Supplementary Method 3: Immobilisation of alkyne/azide modified DNA oligonucleotides

The two patterned regions within the array, with either an alkyne or azide modification, can link to azide or alkyne molecules, respectively, through azide-alkyne Huisgen cycloaddition. For this reaction, we followed a protocol adapted from [10] which links azide molecules dissolved in solution to alkyne modified surfaces and *vice versa*. In our scenario, the DNA strands are modified with either an alkyne or azide end, thus they can be immobilised on the complementary surface groups employing the cited protocol. In this copper-catalyzed reaction, ascorbate is used as reducing agent and a ligand, THPTA, is added to stabilise the cooper ions in the +1 oxidation state. 100 mM potassium phosphate buffer (pH 7) is employed through the whole process. Initially, copper (II) sulphate ($CuSO_4$, Sigma Aldrich) was dissolved in ultrapure (MilliQ) water at a concentration of 50 µM and mixed with 250 µM (dissolved in ultrapure water) ligand THPTA (tris(3-hydroxypropyltriazolylmethyl)amine, Sigma Aldrich). The mixture is added to either 2 µM alkyne or azide modified DNA in potassium phosphate buffer (pH 7). Finally, sodium ascorbate at 1 mM concentration (in ultrapure water) is added to the solution. The solution is then injected through the microfluidic channel. To form the hybridised duplexes, DNA_{azide} and DNA_{alkyne} were injected at 400 nM concentration, using a buffer solution of 1xTE and 1 M NaCl.

Supplementary Method 4: Estimated surface density for the MB layer with optical data

The packing density of the MB layer was estimated by calculating the wavelength shift produced by the presence of an increasing surface concentration of molecules. A number of assumptions were needed in order to perform these simulations. Firstly, a 2D projection of the molecule was estimated to find the average area covered by the each molecule on the sensor's surface.

Secondly, the length of the molecule was also estimated considering a composition of different values for each part of the molecule from the literature, being 3.1 nm. Finally, the refractive index of the MB monolayer was fixed to 1.5 estimated from values within the literature. Simulations were performed using COMSOL multiphysics, in which the resonance shift was simulated against the density of MB molecules on the surface as shown in Supplementary Figure 5. A maximum density coverage of $5x10^{13}$ cm^{-2} was simulated as this was (approximately) the maximum density observed experimentally [3]. A saturation level is also observed at this range of density coverage for this molecule. The value of the surface coverage by the MB molecules is obtained at the intersection of the curve with the value the measured optical shift. In our case, for a shift of 0.58 nm, a surface density of, approximately, 1.5×10^{12} cm^{-2} is obtained, comparing well with values obtained via direct electrochemical measurement.

Supplementary References

[1] Klaassen, D. A unified mobility model for device simulation—I. Model equations and concentration dependence. *Solid State Electron.* **7,** 953-959 (1992).

[2] Klaassen, D. A unified mobility model for device simulation—II. Temperature dependence of carrier mobility and lifetime. *Solid State Electron.* **7,** 961-967 (1992).

[3] Koutsoumpeli, E., Murray, J., Langford, D., Bon, R. S. & Johnson, S. Probing molecular interactions with methylene blue derivatized self-assembled monolayers. *Sens. BioSens. Res.* **6,** 1-6 (2015).

[4] Hoste, J. W., Wequin, S., Claes, T. & Bienstman, P. Conformational analysis of proteins with a dual polarisation silicon microring. *Opt. Express* **22,** 2807-2820 (2014).

[5] Parikha, A. N. and Allarac, D. L. *Handbook of Biofunctional Surfaces.* Pan Stanford Publishing, Boca Raton, 3-29 (2013).

[6] Zadeh, J. N. *et al.* NUPACK: Analysis and design of nucleic acid systems. *J. Comput. Chem.* **32,** 170-173 (2011).

[7] Nedeljkovic, M., Soref, R. & Z. Mashanovich, G. Free-carrier electrorefraction and electroabsorption modulation predictions for silicon over the 1-14um infrarred wavelength range. *IEEE Photon. J.* **3,** 1171-1180 (2011).

[8] Johnson, S. *et. al.* Surface-immobilized peptide aptamers as probe molecules for protein detection. *Anal. Chem.* **80,** 978-983 (2008).

[9] Murray, J. *et al.* Solid phase synthesis of functionalised sam-forming alkanethiol–oligoethyleneglycols. *J. Mater. Chem. B* **2,** 3741-3744 (2014).

[10] I. Presolski, S., Phong Hong, V. & Finn, M. G. Copper-catalyzed azide–alkyne click chemistry for bioconjugation. *Curr Protoc Chem Biol*. **3,** 153-162 (2011).
